# Female patients report comparable results to males after the implantation of an aragonite-based scaffold for the treatment of knee chondral and osteochondral defects: a gender-based analysis of a RCT at 4 years’ follow-up

**DOI:** 10.1186/s10195-025-00829-y

**Published:** 2025-03-13

**Authors:** Elizaveta Kon, Francesca De Caro, Vinod Dasa, Jason M Scopp, Berardo Di Matteo, David Flanigan, Nogah Shabshin, Sabrina Strickland, Nir Altschuler

**Affiliations:** 1 Department of Biomedical Sciences, Humanitas University, Via Rita Levi Montalcini 4, Pieve Emanuele, 20090 Milan, Italy; 2 IRCCS Humanitas Research Center, Via Manzoni 56, 20089 Rozzano, MI Italy; 3 Department of Traumatology, Orthopaedics and Disaster Surgery, Sechenov First Moscow State Medical University (Sechenov University, 6-1 Bolshaya Pirogovskaya St, 119991 Moscow, Russia; 4 Department of Orthopaedics, Istituto di Cura Città di Pavia, Pavia, Italy; 5 Department of Orthopaedic Surgery, Louisiana State University Health Sciences Center, New Orleans, LA USA; 6 Joint Preservation Center, Peninsula Orthopaedic Associates, P.A., Salisbury, MD USA; 7 The Ohio State University Wexner Medical Center, Columbus, OH USA; 8 Division of Musculoskeletal Imaging, Department of Radiology, Penn Musculoskeletal Center, Philadelphia, PA USA; 9 Emek Medical Center, Clalit Healthcare Services, Afula, Israel; 10 Sports Medicine and Shoulder Service, Hospital for Special Surgery, Cornell University Medical College, New York, NY USA; 11 Cartiheal Ltd, Tel Aviv, Israel

**Keywords:** Aragonite, Scaffold, Cartilage, Gender, Microfracture, Randomized

## Abstract

**Background:**

The aim of the study was to provide a gender-based analysis of the results of a large, multi-centre randomized controlled trial (RCT) comparing a novel cell-free aragonite-based scaffold with the standard of care (i.e. debridement/microfractures) for the treatment of chondral/osteochondral defects in knees with or without concurrent osteoarthritis.

**Materials and methods:**

A total of 251 patients were included: 167 patients in the scaffold group and 84 in the control. In the scaffold group, there were 105 males and 59 females, whereas the control group consisted of 51 males and 32 females. Patients were evaluated up to 48 months after the treatment. The primary endpoint was the change from baseline to 48 months in the KOOS overall score. Treatment failures were defined as any secondary invasive intervention, including intra-articular injection or any surgery in the treated joint. All patients underwent magnetic resonance imaging (MRI) at 12 and 24 months to assess the percentage of defect fill after surgery.

**Results:**

Both males and females in the scaffold group achieved significantly better results than controls in any KOOS subscale, as well as in KOOS overall, up to the final 48 months follow-up. Outcomes reported by females were non-inferior to those of males in the implant group. At 24 months’ MRI evaluation, 86.2% of male patients in the scaffold group presented at least 75% defect fill compared with 32.6% in the control group. In the scaffold group, 87.6% of female patients presented at least 75% defect fill, compared with 28% in the control group (*p* < 0.0001 in both cases). Responders’ rate and failure rate were also significantly better in the scaffold group for both males and females.

**Conclusion:**

The aragonite scaffold outperformed the control group at 48 months’ evaluation. The gender-based analysis proved that males and females in the scaffold group presented comparable clinical and radiographical results, both significantly better than their counterparts treated by debridement/microfractures.

*Level of evidence*: I—Randomized controlled trial.

*Trial registration*: Clinicaltrial.gov ID: NCT03299959 (registered on 14 September 2017).

## Introduction

Gender medicine has been gaining increasing attention in the past decades, as an approach to elucidate sex-based differences in the aetiopathogenetic mechanisms of diseases that might also influence the outcomes of specific treatments [1–3]. In the field of orthopaedics, a number of studies have revealed that cartilage damage and degeneration, ultimately leading to osteoarthritis (OA), are linked to some sex-specific pathways [4–6]. In particular, looking at the progression of cartilage pathology, it has been found that different hormonal interactions, anatomical cues and biomechanical features might be responsible for the higher prevalence of OA in the female population [7], which is also affected by more disease severity and disability [5]. Literature has shown that a close interplay exists among all these aspects: some hormones, in particular oestrogens and testosterone, exert a chondroprotective action (thus partially justifying the post-menopausal progression of OA in women), but they also play a role in maintaining muscle trophism, which is often impaired in osteoarthritic joints [8, 9]. Furthermore, the volume of the femoral and patellar cartilage is smaller in females than in males and, over time, females are subject to a faster cartilage loss [10, 11]. Also, biomechanical differences come into play, such as the overall lower congruity in female knee articular surfaces and a different patello-femoral mechanism due to a higher Q angle in women [12, 13]. All these factors may explain the different paths leading to cartilage damage between sexes. This might be particularly relevant in terms of response to treatment, since we might hypothesize gender-based healing mechanisms following cartilage procedures. Current approaches aim at addressing cartilage defects before the onset of severe OA, and in recent years, the indications to treat chondral and osteochondral lesions have expanded to include large defects even in patients affected by concurrent mild-to-moderate OA [14, 15]. Although several different techniques have been developed over time, current research is focusing on regenerative strategies aimed at promoting tissue repair and regeneration by using biomimetic scaffolds. Currently, matrix-assisted autologous chondrocyte transplantation (MACT) and cell-free scaffolds are the most commonly used techniques [16, 17]. Although several studies have reported outcomes on MACT, just a few of them investigated the gender-related outcomes [18], with contrasting results: despite a significant increase in clinical scores documented by most trials in female patients, lower satisfaction rate and higher revision rate than in male patients emerged [19, 20]. Even less investigated are the gender-based differences following implantation of cell-free biomimetic scaffolds [14].

The aim of the present study is to provide a gender-based analysis of the results of a large, multi-centre randomized controlled trial (RCT) comparing a novel cell-free aragonite-based scaffold with the surgical standard of care (i.e. debridement/microfractures) for the treatment of cartilage and osteochondral lesions in knees with or without concurrent knee osteoarthritis. The underlying hypothesis was that scaffold treatment would provide significant and comparable clinical improvement for both male and female patients.

## Materials and methods

### Study design

The present clinical study involved 26 medical centres all over the world and was registered on Clinicaltrial.gov (NCT: XXXXXXX). The protocol was approved by the Food and Drug Administration (FDA) and by the ethical committees/internal review boards of each site. All patients signed an informed consent prior to study inclusion. Patients were randomized to either the aragonite-based scaffold (scaffold group) or debridement/microfractures (control group) in a 2:1 ratio (two patients randomized to scaffold implantation versus one patient randomized to debridement/microfractures). Details on the randomization process and the flowchart of patients’ selection and treatment allocation have been described in a previous paper [21]. Patients were enrolled from September 2017 to November 2019.

### Study device

The Agili-C™ (Cartiheal Ltd, Israel) is a biphasic, cell-free implant consisting of natural inorganic calcium carbonate (aragonite) derived from purified, inorganic coral exoskeleton. This material provides a three-dimensional structure with mechanical properties and high interconnected macro-porosity, required for vascular tissue ingrowth. An extensive description of the physico-chemical properties of the scaffold has been reported in previous trials [22–24].

### Inclusion/exclusion criteria

Main inclusion criteria were as follows: (1) patients aged 21–75 years; (2) presence of up to three joint surface lesion(s), ICRS grade IIIa or above, on the femoral condyles or trochlea; (3) total treatable area 1–7 cm^2^; (4) patients physically and mentally willing and able to comply with the post-operative rehabilitation protocol and scheduled clinical and radiographic visits; and (5) non-responsive to physical therapy for at least 3–4 weeks.

The main exclusion criteria were as follows: (1) KOOS Pain Subscale score at baseline less than 20 or more than 65 (scale: maximum pain, 0; pain free, 100); (2) bony defect depth deeper than 8 mm, according to baseline MRI/X-ray/arthroscopy; (3) articular cartilage lesions in the tibia or the patella, ICRS grade IVa or above; (4) severe osteoarthritis of the index knee, graded 4 according to the Kellgren–Lawrence (KL) score; (5) significant instability of the index knee according to International Knee Documentation Committee (“IKDC”) Knee Examination Form 2000, Grade C (abnormal) or D (severely abnormal); (6) malalignment more than 8 degrees varus or 8 degrees valgus according to standing X-ray; (7) lack of functional remaining meniscus, at least 5 mm rim at the end of the procedure; (8) any known history of intra-articular or osseous infection of the index knee; and (9) uncontained lesion – lack of vital bone wall, at least 2 mm thick, completely surrounding the lesion – based on MRI/X-ray/arthroscopy.

### Surgical treatments

The aragonite-based scaffold implantation technique has been described in previous trials [15, 21]. In brief, the defect site was prepared using a mini-open or open technique, by sequential drilling through the articular surface into the subchondral bone using the dedicated instrument set. Once the preparation was complete, the implant was placed by a press-fit technique so that the top of the implant was at least 2 mm below the articular surface. When multiple implants were used, a bone bridge of at least 5 mm was left between implants to avoid impingement. Implant stability was tested by cyclic bending of the knee while the implant is under direct vision, both before and after tourniquet removal.

Patients randomized to the control group were treated with arthroscopic debridement/microfractures. Debridement consisted of removing the damaged and unstable cartilage fragments from the articular surface, whereas microfracture were performed according to the established technique [25].

In the present study, concurrent procedures, such as osteotomies, partial meniscectomies and meniscal suturing, were allowed, whereas cruciate ligament reconstructions, subtotal meniscectomies and meniscal transplantations were excluded.

### Rehabilitation protocol

The rehabilitation programme includes partial weight bearing for 4 weeks, and then progressive weight bearing to reach full weight bearing at 6 weeks. Continuous-passive-motion (CPM) devices were applied in the early post-operative period and continued for 3 weeks, together with active assisted range of motion (ROM) exercises. Quadriceps isometric sets and electro-stimulation were started initiated immediately post-surgery. Hydrotherapy was advised immediately after suture removal. After approximately 2 months, most patients were able to regain full active ROM and could introduce proprioceptive/balance activities, walking and resistance. Repetitive joint impact activities were allowed after 1 year.

### Outcomes

Patients were evaluated up to 48 months after the treatment. All adverse events occurring during the study period were registered. The primary endpoint was the change from baseline to 48 months in the average KOOS overall score. Furthermore, all the KOOS subscales were analysed. Treatment failures were defined as any secondary invasive intervention, including intra-articular injection or any kind of surgery in the treated joint, regardless if related or unrelated to the original treatment.

All patients underwent MRI at 12 and 24 months to assess the percentage of articular defect fill after surgery. The following MRI protocol was adopted: field of view, 14 cm; slice thickness, 3–3.5 mm; matrix, 512 × 256 (or 384); receiver bandwidth, 80–120 Hx/pixel. Sequences: (a) coronal IW FSE no fatsat; TR ≥ 3000 ms; TE = 30–40 ms; (b) coronal PDW FSE with fatsat; TR ≥ 3000 ms; TE = 10–20 ms; (c) sagittal IW FSE no fatsat; TR ≥ 3000 ms; TE = 30–40 ms; (d) sagittal PDW FSE with fatsat; TR ≥ 3000 ms; TE = 10–20 ms; (e) axial IW FSE no fatsat; TR ≥ 3000 ms; TE = 30-40 ms; (f) axial T2W FSE with fatsat; TR ≥ 3000 ms; TE = ≥ 70 ms; (g) sagittal T1W no fatsat; TR = 600–800; TE = 10–20 ms; (h) oblique PDW FSE with fatsat; TR ≥ 3000 ms; TE = 10–20 ms oriented perpendicularly to the scaffold. Defect fill repair assessment (0–100%) was performed in a blinded manner by an independent radiologist who was an expert in cartilage repair assessment. On each MRI scan, two to three slices located within the implant on a sagittal scan and two to three slices located within the implant on a coronal scan were assessed. For each slice, the degree of cartilage defect volume fill was semi-quantitatively assessed in increments of 25% fill (i.e. 0–24% fill, 25–49% fill, 50–74% and 75–100%). In case of multiple implants/defects, a single range was calculated on the basis of averaging all implants in the same joint.

### Statistical analysis

#### Sample size

The sample size was determined using a “Goldilocks” strategy, as detailed in a previous paper [21]. In brief, seriated interim analyses were to be performed upon enrolment of 250 patients, with increments of 50 up to 500 patients. At each interim analysis, Bayesian predictive probabilities were used to select among the following actions: (1) stop enrolment for anticipated success; (2) stop trial for futility; and (3) continue enrolment. Enrolment was stopped for anticipated success at the first interim analysis with 167 patients randomized to the scaffold group and 84 randomized to control.

#### Outcome analysis

The primary estimand for this study was the treatment group difference in mean improvements from baseline to month 48 in the KOOS overall score. Baseline observation carried forward (BOCF) was applied to study endpoints in case a patient was considered as a failure. Missing data for reasons other than treatment failure were assumed missing at random (MAR) and implicitly imputed through the use of maximum likelihood estimation of a mixed model for repeated measures (MMRM). The MMRM included treatment group, baseline values of the endpoint and all available intermediate values of changes over time. This approach produces unbiased estimates of the treatment effect under the assumption that, conditional on these variables, the likelihood of missing is independent of the distribution of the missing values. The MMRM was used to determine the month 48 treatment group contrast, 95% confidence intervals and *p*-values overall and for males and females separately, for both the scaffold and the control group and for the inter-group difference. Similar analyses were performed stratifying by total lesion size (> 3 cm^2^ versus ≤ 3 cm^2^) and OA status (K-L 0–1 versus 2–3). Demographic and baseline characteristics and secondary outcomes were summarized using descriptive statistics as appropriate.

## Results

### Patients demographics

The study included 251 patients (167 patients in the implant group and 84 patients in the control group). Four patients were excluded from the full analysis set (FAS) (three in the scaffold group and one in the control). As such, 247 patients in total were evaluated at the FAS (164 in the scaffold group and 83 in the control group). In the scaffold group, there were 105 males and 59 females, whereas the control group contained 51 males and 32 females. In both treatment groups, the number of male patients was significantly higher than the number of females. Despite this sample difference, no other significant difference was documented between males and females in baseline features in both scaffold and control groups (Table 1).

**Table 1:** Demographic and baseline features of the included patients, divided by treatment group and by sex

Females	Scaffold group (59 females in total)		Control (32 females in total)	
	*n*	%	*n*	%
Kellgren–Lawrence grade				
0 or 1	32	54.2	12	37.5
2 or 3	27	45.8	20	62.5
Lesion size > 3 cm^2^				
Yes	36	61.0	12	37.5
No	23	39.0	20	62.5
Number of lesions				
1	39	66.1	21	65.6
2	16	27.1	10	31.3
3	4	6.8	1	3.1
Single versus multiple lesions				
Single	39	66.1	21	65.6
Multiple	20	33.9	11	34.4
ICRS grade (worst across lesions)				
Partial chondral (grade 3)	23	39.0	10	31.3
Full thickness (grade 4A)	21	35.6	16	50.0
Osteochondral (grade 4B)	15	25.4	6	18.8
Previous anterior cruciate ligament reconstruction				
Yes	2	3.4	1	3.1
No	57	96.6	31	96.9
Previous meniscectomy				
Yes	10	16.9	7	21.9
No	49	83.1	25	78.1
Concomitant meniscectomy				
Yes	13	22.0	6	18.8
No	46	78.0	26	81.3

Males	Scaffold group (105 males in total)		Control (51 males in total)	
	*n*	%	*n*	%
Kellgren–Lawrence grade				
0 or 1	58	55.2	18	35.3
2 or 3	47	44.8	33	64.7
Lesion size > 3 cm^2^				
Yes	60	57.1	29	56.9
No	45	42.9	22	43.1
Number of lesions				
1	68	64.8	36	70.6
2	35	33.3	11	21.6
3	2	1.9	4	7.8
Single versus multiple lesions				
Single	68	64.8	36	70.6
Multiple	37	35.2	15	29.4
ICRS grade (worst across lesions)				
Partial chondral (grade 3)	23	21.9	17	33.3
Full thickness (grade 4A)	34	32.4	24	47.1
Osteochondral (grade 4B)	48	45.7	10	19.6
Previous anterior cruciate ligament reconstruction				
Yes	11	10.5	6	11.8
No	94	89.5	45	88.2
Previous meniscectomy				
Yes	25	23.8	15	29.4
No	80	76.2	36	70.6
Concomitant meniscectomy				
Yes	34	32.4	12	23.5
No	71	67.6	39	76.5

### Subjective scores

**Fig. 1:**
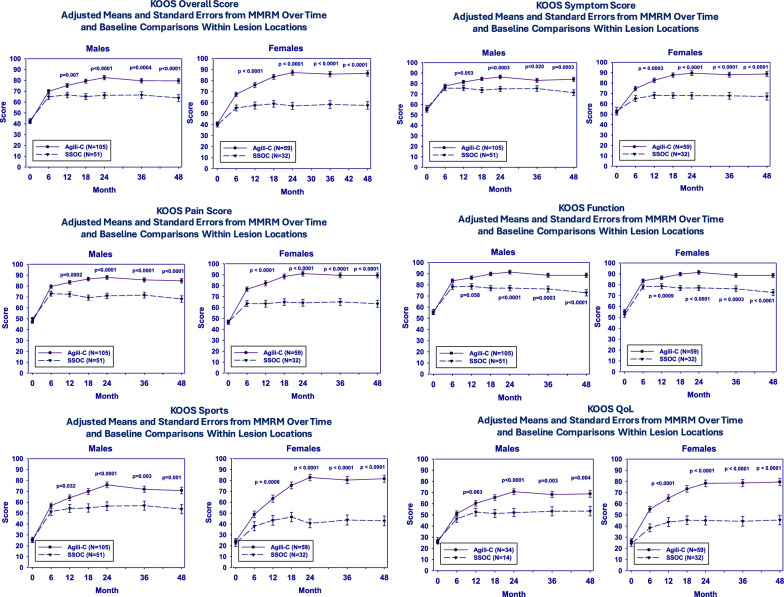
Variation in KOOS score (Overall KOOS + Symptoms, Pain, Function, Sport and Quality of Life subscales) from baseline to month 48, stratified by sex and treatment group.

A gender-oriented comparison was performed to assess the increase in KOOS score from baseline up to the final 48 months’ evaluation: both males and females patients in the scaffold group achieved significantly better results than controls in any KOOS subscale and in KOOS overall, thus proving that the aragonite scaffold outperformed debridement/microfractures at middle-term evaluation, independently of the patient’s gender (Fig. 1).

Interestingly, it was also found that, in the scaffold group, female patients demonstrated greater mean improvements in KOOS “Sport” (*p* = 0.013) and KOOS “Quality of Life” (*p* = 0.025) compared with males.

### Responders’ rate

**Fig. 2:**
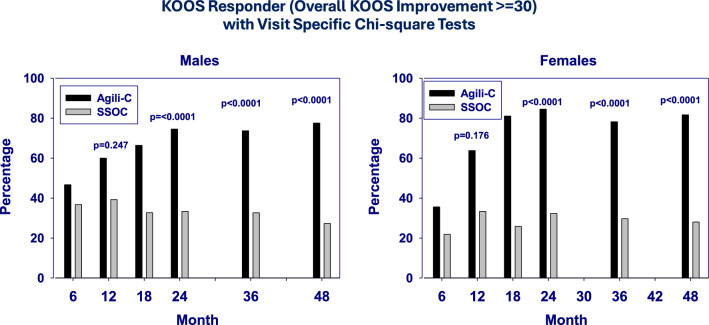
Trend of responders’ rate over time, stratified by sex and treatment group

Responders were defined as patients achieving an at least 30-point increase in the KOOS Overall Score at the various follow-ups compared with baseline. Both male and female patients exhibited a similar pattern: those treated with scaffold implantation showed a higher percentage of responder compared with controls since the first evaluation at 6 months from surgery, and this significant difference was maintained up to 48 months. Looking specifically at the responders’ rate trend in the scaffold group, after 6 months, it was higher in the male subgroup (46.7% versus 35.6%, *p* = 0.02); at 12 months, a comparable responders’ rate was observed between the two subgroups (60.0% in males versus 63.8% in females, *p* value not significant (n.s.)), and then, at the final 4 years’ evaluation, a further increase was observed in both subgroups, without any significant inter-group difference (84.4% for females versus 76.3% for males, *p* value n.s.) (Fig. 2).

### Imaging outcomes

The defect fill assessment was performed at 12 and 24 months’ evaluation. Significantly better radiographic outcomes were achieved in the aragonite scaffold group compared with controls for male and female patients at both timepoints (Table 2). At 24 months, male patients with at least 75% defect fill were 86.2% in the scaffold group compared with 32.6% in the control group, and the percentages for female patients were 92.6% versus 28% in the controls (*p* < 0.0001 in both cases).

**Table 2:** Defect fill evaluated via MRI at 12 and 24 months

Females	Scaffold		Control		*p*-value^1^
	*n*	%	*n*	%	
Month 12 MRI defect fill (%)					
0–24	1	1.8	9	34.6	0.0006
25–49	0	0.0	5	19.2	
50–74	6	10.7	2	7.7	
75–99	34	60.7	4	15.4	
100	15	26.8	6	23.1	
Month 24 MRI defect fill (%)					
0–24	0	0.0	9	36.0	0.0000
25–49	1	1.9	7	28.0	
50–74	3	5.6	2	8.0	
75–99	30	55.6	3	12.0	
100	20	37.0	4	16.0	

Males	Scaffold		Control		*p*-value^1^
	*n*	%	*n*	%	
Month 12 MRI defect fill (%)					
0–24	1	1.0	15	29.4	0.0000
25–49	2	2.0	8	15.7	
50–74	10	9.8	12	23.5	
75–99	73	71.6	13	25.5	
100	16	15.7	3	5.9	
Month 24 MRI defect fill (%)					
0–24	0	0.0	13	30.2	0.0000
25–49	1	1.0	5	11.6	
50–74	13	12.7	11	25.6	
75–99	65	63.7	11	25.6	
100	23	22.5	3	7.0	

Comparison of male and female patients divided by treatment group

^1^*p*-value for Wilcoxon rank-sum test

The intra-group gender-based analysis showed no difference among males and females treated by scaffold implantation both at 12 and 24 months’ evaluations.

### Influencing factors

Patients were stratified according to OA grade, thus comparing those who presented no or minimal sign of OA (Kellgren–Lawrence 0 or 1) with those with mild-to-moderate OA (Kellgren–Lawrence 2 or 3). Independently of the severity of OA, males and females in the scaffold group outperformed their counterparts in the control group (Table 3). Looking at intra-group differences, in the scaffold group, women with mild-to-moderate OA presented a significantly higher increase in KOOS “Overall” from baseline to 48 months follow-up compared with men (47.4 versus 32.3 points, *p* = 0.012; Table 4).

**Table 3:** Subgroup analysis based on the level of OA (KL 0–1 versus KL 2–3) for female and male patients, stratified by treatment group

Females^a^				
Scaffold				
Kellgren–Lawrence score	Mean change from baseline to 48 months	LB of two-sided 95% CI	UB of two-sided 95% CI	*p*-value^2^
0–1	45.64	38.91	52.37	< 0.0001
2–3	47.40	40.07	54.72	< 0.0001
Difference^b^				0.662

Control				
Stratum	Mean change from baseline to 48 months	LB of two-sided 95% CI	UB of two-sided 95% CI	*p*-value^1^
0–1	21.50	10.14	32.86	0.0003
2–3	14.41	5.91	22.92	0.0011
Difference^b^				0.304

Scaffold minus control				
Stratum	Group difference in mean change	LB of two-sided 95% CI	UB of two-sided 95% CI	*p*-value^1^
0–1	24.14	10.94	37.34	0.0005
2–3	32.98	21.76	44.21	< 0.0001

Males^a^				
Scaffold				
Kellgren–Lawrence score	Mean change from baseline to 48 months	LB of two-sided 95% CI	UB of two-sided 95% CI	*p*-value^1^
0–1	41.38	35.64	47.11	< 0.0001
2–3	32.23	25.79	38.68	< 0.0001
Difference^b^				0.035

Control				
Stratum	Mean change from baseline to 48 months	LB of two-sided 95% CI	UB of two-sided 95% CI	*p*-value^1^
0–1	20.19	9.86	30.51	0.0002
2–3	22.29	14.63	29.95	< 0.0001
Difference^b^				0.659

Scaffold minus control				
Stratum	Group difference in mean change	LB of two-sided 95% CI	UB of two-sided 95% CI	*p*-value^1^
0–1	21.19	9.36	33.02	0.0005
2–3	9.94	−0.07	19.95	0.05

*LB* lower bound, *UB* upper bound, *CI* confidence interval

^a^Baseline observation carried forward after treatment failure

^b^Within-group comparison between subgroups at month 48

^1^*p*-value for within-treatment group and subgroup mean changes

**Table 4:** Subgroup analysis based on the lesion size (≤ 3 cm^2^ vs > 3 cm^2^) for female and male patients, stratified by treatment group

Females^a^				
Scaffold				
Stratum	Change from baseline to 48 months	LB of two-sided 95% CI	UB of two-sided 95% CI	*p*-value^1^
> 3 cm^2^	50.21	43.94	56.48	< 0.0001
≤ 3 cm^2^	40.05	32.12	47.99	< 0.0001
Difference^b^				0.051

Control				
Stratum	Change from baseline to 48 months	LB of two-sided 95% CI	UB of two-sided 95% CI	*p*-value^1^
> 3 cm^2^	18.52	7.57	29.47	0.0012
≤ 3 cm^2^	16.41	7.89	24.93	0.0002
Difference^b^				0.998

Scaffold minus control				
Stratum	Group difference in mean change	LB of two-sided 95% CI	UB of two-sided 95% CI	*p*-value^1^
> 3 cm^2^	31.69	19.07	44.32	< 0.0001
≤ 3 cm^2^	23.64	11.97	35.31	0.0005

Males^a^				
Scaffold				
Stratum	Change from baseline to 48 months	LB of two-sided 95% CI	UB of two-sided 95% CI	*p*-value^1^
> 3 cm^2^	36.86	31.19	42.54	< 0.0001
≤ 3 cm^2^	38.12	31.51	44.73	< 0.0001
Difference^b^				0.724

Control				
Stratum	Change from baseline to 48 months	LB of two-sided 95% CI	UB of two-sided 95% CI	*p*-value^1^
> 3 cm^2^	15.34	7.19	23.50	0.0003
≤ 3 cm^2^	29.97	20.46	39.47	< 0.0001
Difference^b^				0.032

Scaffold minus controls				
Stratum	Group difference in mean change	LB of two-sided 95% CI	UB of two-sided 95% CI	*p*-value^1^
> 3 cm^2^	21.52	11.60	31.44	< 0.0001
≤ 3 cm^2^	8.15	−3.41	19.72	0.17

*LB* lower bound, *UB* upper bound, *CI* confidence interval

^a^Baseline observation carried forward after treatment failure

^b^Within-group comparison between subgroups at month 48

^1^*p*-value for within-treatment group and subgroup mean changes

Patients were also stratified according to overall lesion size (i.e. ≤ 3 cm^2^ or > 3 cm^2^). When considering female patients, those treated by scaffold implantation performed significantly better than controls irrespective of lesion’s size. In the male subgroup, instead, the scaffold produced better outcomes than controls in larger lesions (≥ 3 cm^2^) (Table 3).

### Failures

In the scaffold group, 6.7% failure rate was documented for females, compared with 39.4% in the control group (*p* < 0.0001). A similar difference was observed regarding male patients: in the scaffold group, male patients had overall 14% failure rate up to the final 4 years’ evaluation, which was significantly lower than in the control group, where the failure rate was 31.4% (*p* < 0.0001).

### Adverse events

A detailed definition and description of adverse events (AEs) has been provided in a previous paper reporting the results at 2 years’ follow-up [21]. In terms of comparative evaluation, no intra-group difference in AE rate has been reported among males and female both in the scaffold group and in the control group. It is noteworthy that the control group experienced a higher rate of “transient or persisting pain” compared with the scaffold group both for male patients (25.5% in control versus 4.8% in scaffold group, *p* = 0.001) and for female patients (37.5% in control versus 3.4% in scaffold group, *p* < 0.0001).

## Discussion

The main finding of the present gender-based analysis is that the aragonite-based scaffold provides better clinical outcomes than debridement/microfractures at mid-term follow-up in both male and female patients. This has been proven by the following: (1) the comparison of the increase in subjective scores from baseline to the 48 months’ evaluation, which was unilaterally in favour of the scaffold; (2) the higher responders’ rate in the scaffold group for both women and men; (3) the lower failure rate in both sexes in the scaffold group; and (4) the superior radiographic outcomes (defect fill) at 24 months’ evaluation. Beyond these findings, it was noteworthy that females treated by scaffold implantation reported non-inferior results compared with males treated by the same approach.

The results of this gender-based analysis are particularly relevant since they contrast with previous evidence: overall, the available literature highlighted a tendency for lower clinical outcomes in female patients, in particular those receiving MACT, which is the most-studied approach. In fact, although a certain improvement has been demonstrated in female patients following MACT [18], better and longer-lasting results and higher satisfaction rates were described in the male population [20]. Data from the German Cartilage Registry, which included almost 5000 patients treated by different cartilage repair and regeneration techniques [19], highlighted that female patients had better post-operative KOOS increases compared with men but inferior satisfaction rates 24 months after surgery, with higher revision rates. Focusing specifically on MACT, a recent survey from the same registry [26] highlighted that female gender was significantly associated with the risk of re-operations at middle-term follow-up.

Despite these findings, it is worth recognizing the overall paucity of gender-based analyses in the field of cartilage surgery, which might be surprising considering that such procedures are extensively used in routine practice [18]. Although many studies included “simplistic” males versus female analysis as a dichotomous variable, the role of gender cannot be banalized, since it includes a complex network of behavioural, anatomical, biomechanical and molecular features that, considered together, might easily justify an extensive analysis trying to elucidate the reasons of different outcomes between males and females [1, 5, 6]. In this scenario, a hiatus emerges between the vast amount of data existing on the potentially relevant differences between males and females, and their impact reported by interventional studies. Anthropometric studies have shown anatomical differences between male and female knees: first, women have smaller knees both in antero-posterior and medio-lateral dimensions, and therefore, cartilage defects are relatively bigger in female knees owing to a bigger ratio between lesion size and the dimensions of the femoral condyles and tibial plateaux [5, 6]. Furthermore, the thickness of articular cartilage is inferior in women [10], and this should not be necessarily considered a negative point, since scaffold-based treatment aims at restoring the chondral layer, and thus, a thinner healthy cartilage might be more easily replaced by the scaffold [27]. We also know that cartilage metabolism is influenced by hormones, such as oestrogens, which exert a chondroprotective action and whose circulating levels reduce over time in women, thus justifying the higher incidence of cartilage damage in the ageing female population, with inherent onset and progression of symptoms [7–9]. The role of hormones should not be considered only at a local level but, rather, systemically; in fact, they play a significant role in the maintenance of muscles and tendons trophism and influence other biochemical patterns involved in tissue repair and healing [28]. Even pain perception has been hypothesized to be different between men and women, in relation to different expression of pro- and anti-inflammatory molecules occurring over time along the progression of disease [5, 9, 29]. Overall, the entire process of ageing has gender-specific patterns, and therefore, when we look at the influence of age on the outcomes of cartilage treatment, we should remember that age and sex are strictly related [5, 7]. Other relevant sex-specific aspects are biomechanical: for example, the Q angle is higher in women, determining a different biomechanic of the extensor mechanism that could explain the higher prevalence of symptomatic patello-femoral defects in the female population [10, 12, 13]. Women also more frequently present with signs of constitutional laxity, which might explain certain injury and re-injury patterns [30, 31]. Although the list of gender-related influencing factors is very long, unfortunately the clinical trials currently available have failed to elucidate the roles of many of them. This is not necessarily a fault of researchers; evaluating hormones or biomarkers and correlating their levels to the clinical outcomes is not an easy accomplishment and requires the involvement of the lab and an inherent significant increase in expenses. Meanwhile, the assessment of gender-oriented differences in terms of demographics, basal scores, radiographic parameters (limb alignment), lesion features and variations in subjective scores is rarely performed, and matched-paired analysis among male and female patients is occasional in the literature [18]. For these reasons, the role played by gender remains unclear. In the present paper, we tried to shed some light on the potential impact of gender after the implantation of a biphasic, cell-free osteochondral scaffold. Scaffolds are a suitable treatment option for cartilage regeneration: although less diffuse than MACT, they have the advantage of requiring a single surgical step and significantly lower costs since no cell cultivation is required [14, 17]. The aragonite implant used in this trial has been extensively tested in the last decade, at both the pre-clinical and clinical level [15, 21–24, 27], revealing a substantial regenerative potential of the osteochondral unit, sustained by different mechanisms: osteoinduction and osteotransduction for the restoration of the subchondral bone, and direct differentiation of mesenchymal stem cells and cartilage surface progenitor cells to regenerate the chondral layer [27]. The results of animal and ex vivo studies have been backed up in the clinical setting, and the present RCT, whose 2-year results have been previously published [21], allowed the aragonite scaffold to receive the FDA approval owing to its clear superiority over the standard of care (debridement/microfractures), which was again confirmed by the present 48-month follow-up study. The peculiar finding of this gender-based survey is that female patients, following the aragonite scaffold implantation, had non-inferior clinical outcomes compared with men. Interestingly, in some KOOS subscales, female patients presented even better scores than males, and also in terms of failure rate they did particularly well, although it should be noted that women usually are less inclined to re-treatment over time than men, thus justifying this difference in failures. The peculiar feature of the aragonite scaffold is its ability to promote osteochondral regeneration even under stressful joint conditions, i.e. the catabolic environment typical of osteoarthritic knees. The finding that OA female patients, even with larger joint surface defects and concurrent OA, had significant improvements, comparable to those of their male counterpart, might be a game changer in the field of cartilage surgery, since such potential has not been demonstrated by any other technique, where the presence of OA and large defects have always been major concerns to predict failures [13, 32–34]. These positive clinical outcomes have also been supported by MRI results, since both women and men had defect fills superior to those of controls at 24 months. In light of these results, it is possible to state that the scaffold is able to produce beneficial clinical and MRI outcomes both in women and in men for the treatment of knee chondral/osteochondral defects, and a clear superiority emerged for both sexes over the standard of care, particularly in bigger lesions (> 3 cm^2^) and in the presence of osteoarthritic changes.

The strength of the present trial is that it is currently the largest RCT dealing with an osteochondral scaffold, where male and female patients presented comparable basal features in terms of demographics and subjective scores. Furthermore, since no major concurrent procedures were performed, it was possible to evaluate the true clinical potential of the scaffold and compare it with the standard of care (debridement/microfractures).

### Limitations

Some limitations must be acknowledged: the study was primarily powered to detect difference between the aragonite scaffold and the control group, and, in the study protocol, sex was not weighted in the randomization process, thus resulting in a significantly higher number of males in both the scaffold and control groups. This plays a role in the interpretation of results: in fact, although women had better results than men in some outcomes, the smaller sample size recommends caution and does not allow one to affirm the superiority of the scaffold treatment in female patients.

Another flaw of the study is that it did not include data on hormonal status, biomarkers and anatomical/biomechanical parameters of the treated patients for further correlations.

## Conclusion

The aragonite scaffold outperformed the control group at 48 month’s evaluation. The gender-based analysis proved that males and females in the scaffold group presented comparable clinical and radiographical results, both significantly better than their counterparts treated by debridement/microfractures.

## Data Availability

The datasets used and analysed during the current study are available from the corresponding author on reasonable request.

## Abbreviations

AE: Adverse events
BOCF: Baseline observation carried forward
CPM: Continuous passive motion
FAS: Full analysis set
FDA: Food and Drug Administration
FSE: Fast spin echo
ICRS: International Cartilage Repair Society
IKDC: International Knee Documentation Committee
IW FSE: Intermediate-weighted fast spin echo
KL: Kellgren–Lawrence
KOOS: Knee Injury and Osteoarthritis Outcome Score
MACT: Matrix-assisted chondrocyte transplantation
MAR: Missing at random
MMRM: Mixed model for repeated measures
MRI: Magnetic resonance imaging
OA: Osteoarthritis
PD FSE: Proton-density-weighted fast spin echo
RCT: Randomized controlled trial
TE: Echo time
TR: Repetition time
